# Enorme botryomycome du gros orteil

**DOI:** 10.11604/pamj.2016.24.69.8776

**Published:** 2016-05-20

**Authors:** Younes Mhammdi, Mustapha Mahfoud

**Affiliations:** 1 Service de Traumatologie Orthopédie, Hopital Ibn Sina, CHU, Rabat, Maroc

**Keywords:** Botryomycome, ongle incarné, tumeur du gros orteil, Botryomycoma, ingrown nail, tumeur du gros orteil

## Image en médecine

Les auteurs rapportent le cas d'une volumineuse tumeur du gros orteil développé sur un ongle incarné chez un patient de 34 ans. Ce patient a été pris en charge chirurgicalement avec exérèse de la tumeur et cure d'ongle incarné. Il s'agit d'une tumeur exceptionnelle par la taille qu'elle a atteint: 2cm de diamètre, dont la nature a été confirmée sur étude anatomopathologique. Le patient a été revu en consultation régulièrement, la cicatrisation a été obtenue vers la 3eme semaine, pas de récidive avec un recul de 6 mois.

**Figure 1:**
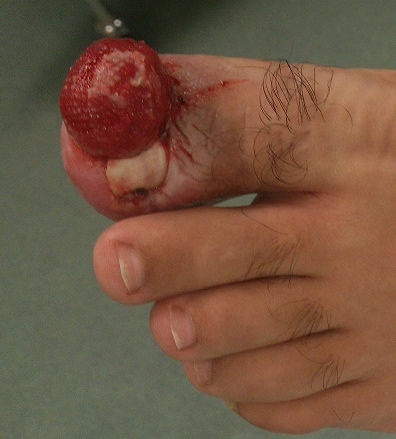
Image montrant une tumeur de 2 cm de diamètre développée sur un ongle incarné du gros orteil

